# Zebra-Fishing for Regenerative Awakening in Mammals

**DOI:** 10.3390/biomedicines9010065

**Published:** 2021-01-12

**Authors:** Laura Massoz, Marie Alice Dupont, Isabelle Manfroid

**Affiliations:** Zebrafish Development and Disease Models Laboratory, GIGA-Stem Cells, University of Liège, B-4000 Liège, Belgium; laura.massoz@doct.uliege.be (L.M.); marie.dupont@uliege.be (M.A.D.)

**Keywords:** zebrafish, regeneration, mammal, liver, pancreas, heart, retina, brain, spinal cord

## Abstract

Regeneration is defined as the ability to regrow an organ or a tissue destroyed by degeneration or injury. Many human degenerative diseases and pathologies, currently incurable, could be cured if functional tissues or cells could be restored. Unfortunately, humans and more generally mammals have limited regenerative capabilities, capacities that are even further declining with age, contrary to simpler organisms. Initially thought to be lost during evolution, several studies have revealed that regenerative mechanisms are still present in mammals but are latent and thus they could be stimulated. To do so there is a pressing need to identify the fundamental mechanisms of regeneration in species able to efficiently regenerate. Thanks to its ability to regenerate most of its organs and tissues, the zebrafish has become a powerful model organism in regenerative biology and has recently engendered a number of studies attesting the validity of awakening the regenerative potential in mammals. In this review we highlight studies, particularly in the liver, pancreas, retina, heart, brain and spinal cord, which have identified conserved regenerative molecular events that proved to be beneficial to restore murine and even human cells and which helped clarify the real clinical translation potential of zebrafish research to mammals.

## 1. Introduction

Humankind has been fascinated with regeneration abilities since the times of the Ancient Greece. In Greek mythology, one of the labors of Hercules was to kill the Hydra, which is able to regrow two heads when one is ablated. In another myth, Prometheus’ liver is renewed every night. However, it was only in the late seventeenth century that scholars paid formal attention to regeneration. Abraham Trembley became a pioneer in this field with his work on fresh water polyps. He described that after cutting a polyp in pieces, each of them was able to regrow an entire organism. He named the polyp “hydra” for its regenerative capacities. After that, regenerative biology had a major influence in the history of biological sciences as it contributed to legitimize biology as an experimental discipline rather than a descriptive science [1].

In recent decades, new technologies such as imaging, genetic engineering and stem cells have enabled the development of regenerative biology, which laid the foundation of a new branch of medicine, i.e., regenerative medicine. Many human diseases and pathologies such as diabetes, Alzheimer’s disease, blindness, heart failure or spine injuries, today incurable, could be cured if functional tissues or cells could be restored by regeneration. However, humans, and more generally mammals, possess limited regenerative capabilities, capacities that even further decline with age. In contrast, invertebrates and phylogenetically primitive vertebrates are able to regenerate full tissues after injury. Even though species with strong regenerative capacities are non-uniformly widespread across the phylogenetic tree, simpler organisms generally perform better in this respect [2]. For this reason, it has been assumed that regenerative potential has been lost during evolution. However, in the last years, several studies have revealed that regenerative mechanisms are still present in mammals but are latent or dormant, and thus it would be possible to stimulate them. This is why elucidating the regenerative mechanisms in competent species is important to permanently cure patients.

Classical models of regeneration are found in invertebrate and vertebrate phylum such as the *hydra*, *planarian*, *drosophila*, *zebrafish*, *axolotl* and *newt*. First exploited to study embryonic development, the zebrafish became in the last 40 years a powerful model organism for deciphering regenerative mechanisms [3]. In 2013, the keyword “regeneration” was the 20th most frequently used in publications using zebrafish [4]. Its success is also due to its fast and external development, the abundant number of eggs and its transparency in the first development stages, making easier the observation of organs and live imaging. Recent technologies, such as CRISPR/Cas9 and TALENs to generate mutant or transgenic lines [5] and high throughput drug screenings [6], have facilitated the study of regeneration in zebrafish. Moreover, its genome is well characterized: 71.4% of the human genes possess at least one or two orthologs in zebrafish and 82% of disease-linked genes listed in the Online Mendelian Inheritance in Man (OMIM) database can be related to at least one zebrafish orthologue [7]. As it has been shown that most of the studied mechanisms in zebrafish implicate the same factors as in mammals, the genetic cascades implicated in a given regenerative process in zebrafish are most likely to be conserved in mammals, rendering possible their manipulation to stimulate regeneration in mammals.

Here we focused on several organs (the heart, liver, pancreas and central nervous system) where research performed in zebrafish clearly helped promote regeneration in murine and human models. These zebrafish studies were selected based on direct evidence of experimental validation in mammalian models (in the same study or in citing references). The overview of these studies also contributes to understanding why the response to tissue damage differs between organs and species and how mechanisms detrimental to regeneration could be overcome.

## 2. Awakening the Regenerative Capacity in Different Organs

### 2.1. The Heart

Unsurprisingly, healthy cardiac function is essential for survival and heart failure remains one of the leading cause of death worldwide [8]. In mammals, even if cardiomyocyte self-renewal does occur, the annual turnover is low, decreasing from 1% to 0.3% between 20 and 75 years old [9], and it is not sufficient to repair injured hearts. Instead, after a myocardial infarction, the damaged myocardium is replaced by fibrotic scar tissue, which tampers cardiac function, ultimately leading to fatal heart failure [10]. By contrast, following a 20% ventricular ablation by resection or cryoinjury, the zebrafish fully regenerates a functional myocardium within a few months without scarring, even at the adult stage (Table 1) [10,11,12,13,14,15]. Although this regenerative capacity is also observed in neonatal mice, in contrast to zebrafish, it is lost after the first week of postnatal life [16].

Using cardiomyocyte lineage tracing systems in adult fish and neonatal mice, regenerated cardiomyocytes were shown to derive from dedifferentiation of pre-existing mature cardiomyocytes followed by proliferation and redifferentiation, rather than from progenitor or stem cells [16,17]. The ploidy of cardiomyocytes is one of the major differences between adult zebrafish and mice. Adult zebrafish cardiomyocytes are mainly diploid and mononucleated with a high proliferative potential during regeneration [18], whereas the non-regenerative myocardium of adult rodents and humans is largely composed of polyploid mono- or binucleated cardiomyocytes [19,20,21]. Polyploidy has been proposed to account for the decreased regenerative potential of these species [22].

The RhoGEF Ect2 is required for cytokinesis initiation [23] and its expression in murine cardiomyocytes decreases during the first week of postnatal life [18] correlating with the binucleation event and the loss of regenerative ability [16,24]. However, its expression remains high in zebrafish [18]. Using a transgenic line inhibiting *ect2*, Gonzalez-Rosa et al. managed to induce cardiomyocyte polyploidization in zebrafish. After heart injury, they observed that an excess of 50% of polyploid cardiomyocytes dampens the proliferation of remaining cells, and thus the regeneration of the organ, while it induces a persistent scarring. This highlighted an inverse correlation between the percentage of polyploid cells and the regeneration ability of the heart [18]. The mobilization of diploid instead of polyploid cardiomyocytes in mammals, by maintaining the expression of *ect2*, would offer a therapeutic alternative to stimulate the proliferation of cardiac cells and heart regeneration.

**Table 1:** Overview of injury models presented in the review.

Organ	Model Organism	Injury Model	Type of Injury	Mechanism of Regeneration	Characteristics	References
Heart	Zebrafish	Ventricular resection	Surgical	Proliferation from pre-existing myocytes	-	[11]
		Cryoinjury	Surgical	Proliferation from pre-existing myocytes	Clinically relevant to mammalian infarcts with massive cell death	[13]
	Mouse	Ventricular resection	Surgical	Proliferation from pre-existing myocytes	Fully regenerates a functional myocardium in 1–2 months without scarring	[11,12]
		Myocardial infarction	Surgical	Proliferation from pre-existing myocytes	Left anterior descending coronary artery occluded with a nylon suture	[25]
Liver	Zebrafish	Partial hepatectomy	Surgical	Hepatocyte-driven	Clinically relevant	[26]
		APAP overdose	Chemical	BEC-driven regeneration	Paracetamol overdose	[26]
		Nitroreductase (NTR)-mediated ablation	Genetic/Chemical	Hepatocyte-driven BEC-driven regeneration	*Tg(fabp10a:CFP-NTR)*	[27,28,29]
	Mouse	CDE diet	Chemical	BEC-driven regeneration	Ethionine, a toxic analog of methionine, in association with choline deficiency, leads to hepatocyte death and liver inflammation	[30]
		*Ctnnb1* hepatocyte KO	Genetic	BEC-driven regeneration	Represses hepatocyte proliferation—in combination with an injury model	[29]
		*Mdm2* deletion (hepatocyte-specific)	Genetic	BEC-driven regeneration	AhCreMdm2flox/floxInducible, repress hepatocyte proliferation	[30]
Beta cell/Pancreas	Zebrafish	NTR-mediated ablation	Genetic/Chemical	Beta cell proliferation;alpha cell transdifferentiation; Neogenesis from ductal progenitors	*Tg(ins:CFP-NTR)* In cells expressing NTR, reduces non-toxic pro-drug into cytotoxic products causing targeted cell apoptosis	[31,32,33,34]
	Mouse	Pancreatic Duct Ligation (PDL)	Surgical	Neogenesis from ductal progenitors	Induces acinar cell death and acute inflammation without destruction of beta cells	[35,36,37]
		Streptozotocin (STZ)	Chemical	Beta cell proliferation; Neogenesis from ductal progenitors	Toxic glucose analogue that enters into beta cells via the GLUT2 transporter causing their death	[31,38]
		Diphtheria Toxin Analogue (DTA)	Genetic/Chemical	Alpha cell transdifferentiation (adult only) Delta cell transdifferentiation (neonatal only)	*Tg(RIP:DTR)* The toxin enters in cells expressing the DTR and inhibits protein synthesis, leading to cell apoptosis. Here targeted in beta cells with the Rat Insulin Promoter (RIP).	[39,40]
Spinal Cord	Zebrafish	Spinal cord transection	Surgical	Glial bridge	Complete cutting of the vertebral column	[41]
	Mouse	Laminectomy and spinal cord hemisection	Surgical	-	Hemisection leading to complete paralysis of the ipsilateral limb	[42]
Brain	Zebrafish	Stab-lesion assay	Surgical	Regeneration from radial cells	Injury in the telencephalon parenchyma	[43]
		B42 mediated injury	Surgical/Chemical	Regeneration from radial cells	Alzheimer’s-disease-like	[44]
	Mouse	AD-like model	Genetic	No regeneration	*APP/PS1dE9* transgenic	[45]
Retina	Zebrafish	Needle poke	Surgical	From Muller cells	-	[46]
		Optic nerve lesion	Surgical	From Muller cells	-	[47]
	Mouse	NMDA	Chemical	From Muller cells	-	[46,48]
		Excessive light	Surgical	From Muller cells	-	[46]
		AD-like model	Genetic	No regeneration	APP/PS1dE9 transgenic	[45]

In order to identify other mechanisms underlying heart regeneration in adult zebrafish and potentially conserved but dormant mechanisms in mammals, Aguirre and colleagues focused on the microRNAs (miRNAs) differentially regulated after amputation of the ventricular apex in adult zebrafish [25]. They identified miR99/100 and let-7a/c that are downregulated during regeneration. These miRs are known to be implicated in proliferation, chromatin remodeling and morphogenesis, including cardiomyogenesis. Downregulation of miR99/100 during the cardiac regenerative process in zebrafish allows a significant de-repression of their targets *fntb* and *smarca5* in cardiomyocytes, associated with increased cell cycle entry. By contrast, in adult mouse and in human heart tissue, the expression of miR-99/100 stays high after injury, inhibiting the expression of *Fntb* and *Smarca5*. Silencing of miR99/100 or let-7a/c in isolated primary murine adult cardiomyocytes or in murine organotypic slices induced cardiomyocyte dedifferentiation and the acquisition of a proliferative phenotype similar to what was observed in zebrafish. Similar results were obtained in vivo by intracardiac injection of anti-miR-99/100 and anti-Let-7a/c in a murine model of myocardial infarction. More importantly, this led to an improvement of functional heart parameters after 15 days and to the reduction of fibrotic scarring and of the infarct size compared to scrambled controls. Of note, the dedifferentiation observed after miR99/100 downregulation in mammalian cardiomyocytes is limited to the mononucleated cells. Either the polyploid cardimoyocytes are able to convert to a mononucleated state or the mononucleated cardiomyocyte population is more responsive to the regenerative pathway [25]. It would be interesting to answer this question in order to find the best strategy to induce cardiomyocyte proliferation in mammals. In conclusion, the limited cardiac regeneration in mice is at least due to the failure to modulate the miR99/100 and let-7a/c/FNTB and SMARCA5 axis and anti-miR delivery can reactivate this dormant pathway in mammals [25,49].

In the same way, comparison of gene and miRNA profiling of injured zebrafish and mouse adult hearts identified miR-26a [50]. miR-26a represses expression of *ezh2*, a key component of the polycomb repressive complex involved in the methylation of histone H3K27 that is implicated in cardiomyocyte proliferation and in the maintenance of cardiac identity in mice. After ventricular resection in zebrafish, *ezh2* expression is induced due to the downregulation of miR-26a whereas, in the murine heart, miR-26a expression remains high after injury and maintains inhibition of *Ezh2*. Knock-down of miR26a in neonatal mice via injection of anti-miR-26a oligonugleotides increased expression of *Ezh2* and augmented the number of proliferating cardiomyocytes [50].

Together, miR-99/100 and miR-26a are downregulated during the regenerative process in zebrafish whereas their expression is high in adult mice [25,50]. As their expression can be inhibited by antagomir therapy in the mammals, miRNAs could constitute clinical targets to stimulate cardiac regeneration [25,49,50].

Other transcriptomic analyses from adult zebrafish have shown that leptin B (*lepB*), a paralog of mammalian leptin, is induced in the regenerating tail fin and heart [51]. By epigenetic profiling, Kang et al. have identified a short sequence upstream the *lepB* promoter, called *lepb-linked enhancer* (LEN), which acquires H3K27ac marks and open chromatin marks during regeneration [51]. Moreover, the authors showed that LEN can direct regeneration-activated gene expression not only from *lepB* but also from different promoters such as *cmlc2* (cardiomyocytes) or *α-cry* (lens). They exploited this LEN sequence to overexpress *neuregulin 1* (*nrg1*), known to be implicated in cardiomyocyte proliferation and regeneration [52], in adult zebrafish via transgenesis using a promoter combining LEN and the *lepB* minimal promoter. After ventricular resection, these fish strongly activated *nrg1* expression at the injured area and exacerbated cardiomyocyte proliferation. In contrast, control fish did not induce expression of *nrg1* under the *lepB* minimal promoter only without the LEN sequence, showing that LEN can modulate heart regeneration. Even if the LEN sequence is poorly conserved in mammals, LEN-hsp68::lacZ transgenic mice where the zebrafish LEN was fused to the murine *hsp68* promoter revealed injury-dependent LEN activity in wounds after heart resection or even digit amputation in neonates [51]. This result shows that mammalian gene regulatory networks have the potential to activate zebrafish LEN enhancer and to enable injury-induced expression in mice [53], suggesting that similar constructs could be designed to stimulate timely regeneration of different organs in mammals. It remains to determine whether overexpression of *Nrg1*, or of other positive regulators, under the LEN promoter could give similar results.

### 2.2. The Liver

Despite their poor regenerative capabilities, mammals are able to efficiently regrow their liver. After partial hepatectomy or mild injury, liver regeneration is mainly achieved by proliferation of pre-existing hepatocytes. However, this process is impaired after acute injury or in hepatic chronic diseases such as liver cirrhosis, viral hepatitis and liver cancer. These diseases are characterized by inflammation, fibrosis and exhaustion of the proliferative potential and finally the death of the hepatocytes. In these situations, activation and expansion of biliary ductular cells, the so-called “ductular response”, takes place. Oval cells have been observed in mammals next to these ducts [54] and it has been hypothesized that they could represent liver progenitors deriving from ducts able to restore hepatocytes. Many rodent models of chronic liver injury have been developed to study this process (Table 1). One category of models involves hepatotoxins such as ethionine, CCl4 or N-acetyl-p-aminophenol (APAP), (also called paracetamol or acetaminophen), which are repeatedly injected or delivered in association with specific pro-inflammatory diets, causing chronic death of hepatocytes. The second category of models consists of mutant models (*Mdm2*, *Ctnnb1*, *Itgb1*, *p21/Cdkn1a*) with impaired hepatocyte proliferation. Models to study liver regeneration from the ducts usually combine chronic hepatocyte injury and repression of replication. Previously the subject of controversy, mainly owing to the diversity of the models, there is now strong evidence that biliary epithelial cells (BECs), also known as cholangiocytes, are able to dedifferentiate into liver progenitor cells (LPCs), or oval cells, when hepatocyte-driven regeneration is compromised [30]. These LPCs are bipotent progenitors able to redifferentiate into BECs or hepatocytes. It is of utmost clinical importance to identify the molecular regulation of this process, still not yet fully understood, to improve liver regrowth in chronic hepatic disease patients. This particular topic has been recently reviewed [55,56].

The zebrafish can efficiently replenish its liver with new hepatocytes through both hepatocyte-driven (i.e., replication) or BEC-driven regeneration, providing a valuable model to decipher the mechanisms of both types of liver regeneration. A chemical screening performed in the zebrafish *Tg(fabp10a:CFP-NTR)* line, based on nitroreductase (NTR)-mediated near-total ablation of hepatocytes, pinpointed that the bromodomain and extra-terminal proteins (BET) are required for BEC-driven regeneration [28]. BETs recognize lysine acetylation in histones and other transcription factors, thereby positively or negatively regulating transcription. They mediate different steps of BEC-driven regeneration in zebrafish, BEC dedifferentiation into LPC, proliferation of LPC and redifferentiation into new hepatocytes and their maturation [28]. In addition, BET proteins also promote hepatocyte-driven liver regeneration in a zebrafish liver injury model of paracetamol (APAP) overdose (Table 1) [26]. Importantly, the requirement for BET proteins in both types of liver regeneration is conserved in mice. In the choline-deficient ethionine-supplemented CDE-diet mouse model of chronic liver injury that induces BEC-driven regeneration, BET proteins are required for activation of LPC [28]. In mice after partial hepatectomy, BET proteins are required for hepatocyte proliferation [26]. These data are of high clinical relevance as the same BET inhibitor, JQ1, has been used in a clinical trial for cancer therapy, including liver cancer. The authors stressed that, even though such drugs could be beneficial in this specific context, they would also inhibit liver regeneration, thereby limiting their therapeutic use [26]. Given the importance of BET proteins as epigenetic regulators, a second chemical screen with a library of compounds targeting epigenetic factors has been conducted with the same zebrafish NTR-mediated liver ablation model [29]. This screening identified the histone deacetylase HDAC1 as a potential regulator of BEC-driven regeneration. HDAC1 was already known to be involved in liver regeneration in mouse models of hepatocyte-driven regeneration [57]. Ko et al. 2019 showed that *hdac1* regulates LPC differentiation into hepatocytes and BECs during BEC-driven regeneration in zebrafish [29]. More exactly, the loss of *hdac1* impairs LPC differentiation into hepatocytes by increasing the expression of *sox9b*, and into BECS via the increased expression of *cdk8*, a negative regulator of Notch signaling. Administration of the HDAC1 inhibitor MS-275 to a mouse model of chronic liver injury combining hepatocyte-specific loss of *ctnnb1* (β-catenin) and a choline-deficient, methionine-supplemented diet impairs differentiation of LPCs into hepatocytes [29]. Interestingly, *HDAC1* is expressed in liver tissues from patients with cirrhosis, suggesting a conserved role of *Hdac1* from zebrafish to human in LPC differentiation [29].

Another approach to decipher regenerative molecular mechanisms is the identification of candidates by RNA sequencing. Using this approach and the zebrafish NTR-mediated ablation model, the Bone Morphogenetic Protein (BMP) pathway was found to be modulated during liver regeneration [27] and BMP inhibition impaired BEC-driven regeneration. Based on these findings, BMP2 treatment was shown to increase the differentiation of a murine liver progenitor cell line into hepatocytes in vitro. To conclude, screenings conducted in zebrafish enabled us to identify mechanisms of both hepatocyte- and BEC-driven liver regeneration, which seems conserved in mammals.

### 2.3. The Pancreas

Diabetes is a leading health issue worldwide with an incidence of 1 out of 11 people, and causes 1.5 million of deaths per year according to the WHO. The disease is characterized by a dysfunction of blood glucose regulation and various consequent life threatening health conditions. In type 1 diabetes (T1D) or in late stages of type 2 diabetes (T2D), the insulin-producing beta cell mass is dramatically decreased, resulting in a lack of insulin. Besides therapeutic strategies to preserve the beta cell mass and its function and to improve insulin treatments, beta cell regeneration constitutes a promising alternative to replenish the pancreas with functional beta cells. This process is extensively studied in mice, using a model of pancreas injuries. In rodents, the main models of beta cell regeneration consist of injections of a toxic glucose analogue streptozotocin (STZ), expression of the diphtheria toxin A (DTA) suicide transgene, and the pancreatic duct ligation (PDL) model which is characterized by high levels of inflammation in the pancreas but no destruction of beta cells per se (Table 1). Using these models, mice revealed a certain plasticity of mammalian pancreatic cells despite the poor regenerative capacity of mammals. Besides replication of remaining beta cells [58], neogenesis can proceed from alpha cells [40], delta cells [39] or acinar cells [59]. Duct-associated pancreatic progenitors have also been proposed [37,38] even though this source is under controversy [35,36]. These studies underline the importance of the injury model and of age in regeneration efficiency and cellular origin of new beta cells.

To get new insights into beta cell regeneration and to overcome the limited regeneration ability of rodent models, researchers have exploited the zebrafish model. One of the strategies was to identify pharmacological compounds able to enhance beta cell proliferation. Several groups performed medium or high-throughput drug screenings using zebrafish larvae and the inducible NTR-mediated ablation model [31,32,60]. Two independent studies discovered that drugs stimulating the production of cAMP promote beta cell regeneration by proliferation. One class of compounds activates the adenosine/cAMP pathway and promotes beta cell proliferation after beta cell ablation [31], of which the more potent is the *50-N-ethylcarboxamidoadenosine* (NECA), an adenosine agonist activating GPCR signaling. The other study identified the TBK1 and IKKε inhibitor *(E)-3-(3-phenylbenzo[c]isoxazol-5-yl) acrylic acid* (PIAA), which appeared to activate the cAMP-PKA-mTOR pathway leading to increased beta cell proliferation after ablation [32]. Importantly, both drugs, NECA and PIAA, also increased beta cell proliferation in mammalian ex vivo models, NECA being validated in mouse islets and PIAA in both rat and human islets. NECA and PIAA were also able to enhance beta cell regeneration in vivo in STZ-treated mice. Moreover, these drugs led to functional improvement by lowering glycemia in mice [31,32]. By coupling the advantage of assessing the effect of various compounds on a given phenotype (here beta cell regeneration) with toxicological assays, the zebrafish allows not only the pinpointing of the adenosine pathway but also the identification of, among the numerous cAMP modulators, the non-toxic compounds most promising for further clinical studies. Validations in human models are particularly critical in the context of beta cell replication as adult human beta cells are extremely resistant to cell cycle re-entry compared to mice [61]. In addition, beta cell replication is inversely correlated with functional maturation, thus such a strategy should be used with caution.

Besides beta cell proliferation, other pancreatic cells can give rise to new beta cells. The glucagon-producing alpha cells are able to transdifferentiate into beta cells in various mouse models [40], though the regeneration is very slow and low, as well as in the zebrafish NTR model [34]. After a transcriptomic profiling by microarray of zebrafish islets isolated during regeneration following NTR-mediated ablation, secreted proteins were selected as candidate enhancers of beta cell regeneration [33]. One of them, the insulin-like growth factor (Igf) binding-protein 1 (*igfbp1*), was shown to increase transdifferentiation of alpha cells into beta cells when overexpressed by transgenesis, leading to potentiation of beta cell regeneration and accelerated restoration of normoglycemia. Furthermore, IGFBP1 could also promote alpha cell transdifferentiation in mouse and human islets ex vivo. Since *igfbp1* is known to be repressed by insulin, the study also showed that patients with insulin resistance have a lower level of IGFBP1 in their blood while those with a high level of IGFBP1 have a lower risk to develop T2D [33]. In T1D or in late stages of T2D, when the beta cell mass is reduced, the level of IGFBP1 is elevated due to the lack of insulin [62]. These observations demonstrate that IGFBP1 could be a potential biomarker for insulin resistance/diabetes in addition to be a good candidate for beta cell regeneration in (pre)clinical studies.

Another axis of regeneration is to harness pancreatic progenitors. As endocrine cells arise from the pancreatic ductal tree during the development, it has been hypothesized that progenitors could still be associated to the pancreatic ducts in adults. Although beta cell neogenesis from ducts in the adult is under controversy in mammals [35,36,37,38,63], it is well established in the zebrafish [64,65,66]. A drug screening performed without regeneration but in conditions boosting beta cell formation from the ducts in zebrafish larvae, pinpointed two inhibitors of CDK5, roscovitine and DRF, as enhancers of beta cell differentiation from ductal-associated progenitors [67]. Inhibition of *cdk5* has then been shown to stimulate regeneration after beta cell ablation. This finding has been validated in mouse embryonic pancreatic explants, in human iPSCs and in vivo in the PDL mouse model, though glycemia and glucose tolerance were not improved. To summarize, with one unique zebrafish model (the NTR-mediated ablation) it was possible to explore beta cell regeneration from different cellular origins and to identify pharmacological compounds and signaling pathways able to promote beta cell regeneration in mammals.

### 2.4. The Central Nervous System (CNS)

The central nervous system (CNS) is composed of two main cell types: neurons and glial cells. Neuronal cells are the basic functional units of the CNS capable of sensing and transmitting information via electrochemical pulses. The main roles of glial cells are to maintain homeostasis and to support and protect neurons. The earliest glial cells formed during embryonic development are the radial cells. These cells act as neuronal progenitors and thus give rise to neurons and intermediate progenitors. However, their neurogenic capacity decreases while they differentiate into star-shaped astrocytes. In the adult mammalian brain, the neurogenic capacity of the glia is restricted to few specific regions, called neurogenic niches, where the astroglia can still give rise to a few new neurons. Some of the astroglial cells in these neurogenic niches are considered as neural stem cells. Although it is possible to observe star-shaped cells in zebrafish, there are no clearly defined astroglial cells in this species, and glial cells in zebrafish retain their radial identity through life. Thus these radial/astroglial cells have important neurogenic capacities. These cells can give rise to new neurons not only in the neurogenic niches but more broadly in the CNS [68], and they constitute the basis of regeneration in the CNS, i.e., the spinal cord, the brain and the retina.

#### 2.4.1. The Spinal Cord

Spinal cord injury in mammals is followed by formation of a dense and heterogenous network composed of hypertrophic stellate astrocyte gliosis, fibroblasts and inflammatory immune cells, called the glial scar. This scar establishes a mechanical and impenetrable barrier impeding the regeneration of severed axons and repair of neuronal circuits [69,70,71]. In zebrafish, complete transection of the spinal cord (Table 1) results in tissue discontinuity and loss of glial and axonal connections. Then, glial cells proliferate and migrate to the injured area and acquire a bipolar and elongated morphology, forming a glial bridge. This allows, by 5 weeks, the regeneration of axons from viable neurons across the lesion site and their reconnection to the central canal, and fish recover their normal swimming behavior [72]. Notably, unlike mammals, this regeneration process is not accompanied by formation of a scar [72,73,74]. The formation of this bridge results from differential regulations compared to mammals allowing the presence of a permissive pro-regenerative microenvironment in zebrafish. One of these key regulations is a dynamic transient inflammatory response in zebrafish. Indeed, 2–3 days after spinal cord injury, the initially proinflammatory environment switches to an anti-inflammatory one with notably the presence of M2 macrophages, whereas, in mammals, pro-inflammatory macrophages persist at the wound site for a long time after injury [74].

A key regulator of the formation of the glial bridge is Fibroblast Growth Factor (FGF) signaling. Indeed, in zebrafish, the expression of several FGF ligands (*Fgf2*, *3*, *8*) and their downstream targets (*spry4*, *pea3* and *erm*) are increased at the injured site [72,75]. Using several models of gain or loss of function, Goldshmit and colleagues have examined the role of FGF during spinal cord regeneration in a series of studies from zebrafish to in vitro and in vivo mammalian models [42,72,76]. They first established in zebrafish that the FGF signaling is necessary for the formation of the glial bridge and for axonal regeneration. Next, they showed that in vitro treatment of primate primary astrocytes with recombinant human Fgf2 (hFgf2) recapitulated some of the characteristics of zebrafish glia cells during spinal cord regeneration such as acquisition of a bipolar elongated shape [72]. In mice, hFgf2 injection after spinal cord hemisection promotes formation of a glial bridge rather than a scar, allowing the growth of neurites and axonal regeneration through the lesion site. Mice injected with hFgf2 also displayed reduced inflammation, less macrophage and microglia activation and reduced leukocyte infiltration [42]. Moreover, these mice showed an improved functional recovery compared to control animals. These results are consistent with previous studies showing that acidic FGF and FGF2 are implicated in locomotor recovery in rodents [77,78]. Interestingly, similar observations were made with endogenous increase of FGF signaling in *spry4*^−/−^ mutant mice, *spry4* being a feedback inhibitor of this pathway [76].

One promising cellular therapy following a spinal cord injury is the transplantation of stem cells directly into the injured site. Dental pulp cells (DPC) are composed of many types of stem cells and their transplantation induced an enhanced improvement of the functional recovery in a rodent spinal cord injury model compared to bone marrow-derived stromal cells transplantation [79]. These results are even more promising when human DPC are pretreated with FGF2 for several consecutive serial passages and then directly transplanted into the injury site with, notably, an improvement of axonal regeneration and of the locomotor recovery of the hind limbs by improving the survival rate of DPC at the lesion site [80].

Altogether, these results demonstrate a conserved pro-regenerative role for the FGF signaling in the formation of the glial bridge and, hence, in axonal regeneration in the spinal cord.

#### 2.4.2. The Brain

Aging, brain injury or neurodegenerative conditions such as Alzheimer’s disease (AD) and Parkinson’s disease cause a major loss of neural cells. After brain injury, glial cells present in the neurogenic niches have the potential to proliferate. However, a reactive gliosis also occurs, producing a glial scar that inhibits this proliferation and hampers neurogenesis. The zebrafish brain has an incredible capacity of regeneration that can be partially explained by its numerous neurogenic niches and by the capacity of radial cells in the parenchyma to form new neurons. Depending on the type of injury and its localization and severity, different mechanisms of regeneration can be activated. This topic has been recently reviewed [68,81,82]. In this section, we focus on two different models of zebrafish brain injury/neurodegeneration that lead to mechanistic translation in mammalian models. In the zebrafish stab lesion assay (Table 1), the parenchyma of the telencephalon is surgically injured but leaves the neurogenic niches intact, allowing radial cells to proliferate, migrate and generate new neurons [83]. Kizil et al. 2012b showed that the expression of the zinc finger transcription factor Gata3 is induced in radial cells in response to injury [43] where its activity is necessary to properly activate their proliferation, neurogenesis and to promote migration of the newborn neurons, specifically in an injury context [43]. Human/mouse astrocytes fail to induce *Gata3* in response to injury. To mimic injury conditions, scratches have been performed in 2D and 3D cultures of human astrocytes. However, though *Gata3* delivery increased the number of neuronal progenitors, they could not achieve neurogenesis [84]. These results show that *Gata3* enhances the neurogenic potential of human astrocytes but is not sufficient.

To study the mechanisms of brain plasticity in response to neurodegeneration, a zebrafish model of Alzheimer’s-disease-like (AD) has been developed [44] (Table 1). A hallmark of AD is the accumulation of β-amyloid Ab42 aggregates in the brain. Injection of Ab42 peptides coupled with a cell peptide transporter (transportan) into the zebrafish brain lead to neurodegeneration [44]. In contrast to mammals, neurodegeneration triggers radial/astroglia cell proliferation and neurogenesis in zebrafish. Transcriptomic profiling of this zebrafish model showed that *gata3* does not seem to be involved but pinpointed immune signaling pathways upregulated in response to Ab42-mediated neurodegeneration. This uncovered the specific upregulation of the anti-inflammatory interleukin-4 (IL4)/STAT6 pathway and its beneficial action on glial cell proliferation in the AD-like model. In contrast, this pathway is not activated in mammals. Furthermore, IL4 overexpression in healthy zebrafish could increase brain progenitor proliferation and neurogenesis [44]. Papadimitriou et al. 2018 later developed a 3D-culture model of human astrocytes and neural stem cells and examined the effect of IL4 as these cells naturally express the IL4 receptor. Interestingly, the inhibitory effect of Ab42 peptide on proliferation capacities could be rescued by treatment with IL4 [85]. However, in an in vivo mouse model of AD, the expression of the IL4 receptor could not be detected in astrocytes [45] and its artificial delivery led to astrocyte death [45]. The authors hypothesized that the mammalian brain evolved to avoid hyper-proliferation by establishing a non-permissive environment for cells expressing the IL4 receptor. 

To summarize, thanks to two different zebrafish models of brain regeneration, mediators of brain plasticity with favorable potential in mammalian models were identified though the complexity of the mammalian brain, which has evolved rigid barriers to repress regeneration in order to, presumably, avoid tumorigenesis.

#### 2.4.3. The Retina

Photoreceptor death characterizes retinal degeneration and eye diseases like diabetic retinopathy, retinitis pigmentosa or glaucoma, leading to loss of vision and even to complete and untreatable blindness. A promising strategy to restore sight would be to activate endogenous regeneration of photoreceptors within the retina. Exploiting regenerative capacities of amphibians and fish, researchers revealed several cellular sources to regrow new photoreceptors. Among them, we can cite the retinal pigment epithelium (in amphibians but not in fish), the ciliary margin (the region which contains the retinal stem cells in fish and amphibians) and the Müller glia [86]. The Müller cells (MCs) constitute the major glial cells spread through the entire retina and are conserved from fish to mammals. Their function is to maintain retinal homeostasis and structure. During retinal development, the MCs are the latest cells to arise from retinal multipotent progenitors. MCs share molecular signatures with late retinal progenitor cells [87], leading to the hypothesis that MCs could be progenitors with a glial function. In response to retinal injury, MCs undergo reactive gliosis, i.e., change in morphology, dedifferentiation and proliferation [88,89]. However, this proliferation is rapidly inhibited in mammals, resulting in scar formation and preventing regeneration. On the other hand, zebrafish MCs can differentiate into new retinal neurons after replication [47] and restore vision. Assuming that this regenerative capacity is present in mammals but dormant, researchers focused on key factors specifically expressed or induced in zebrafish but not in mammals. The most tangible example is the case of achaete scute-like family bHLH transcription factor 1a (ASCL1a). In response to surgical injury, *ascl1a* expression is induced in the zebrafish retina and is necessary for MC proliferation and thus regeneration [90], while *Ascl1* is not expressed in the mammalian retina. In order to test if *Ascl1* expression can stimulate the neurogenic potential of mammalian MCs, *Ascl1* has been overexpressed in ex vivo explants of mice MCs, which enabled their dedifferentiation into retinal progenitors [91]. Furthermore, while *Ascl1* expression driven in vivo by transgenesis in mice retina did not affect the uninjured retina, it could activate regeneration after injury induced by N-methyl D-aspartate (NMDA) or excessive light [92] (Table 1). However, only juvenile mice were able to generate new retinal neurons, showing that *Ascl1* is important but not sufficient to induce retinal regeneration in adult mammals [92]. Epigenetic regulations were proposed to underlie the age-dependent regenerative capacities as the *Ascl1* target genes are accessible in juvenile MCs but less accessible in adult MCs [92]. Supporting this hypothesis, *Ascl1* overexpression in MCs combined with an eraser of epigenetic marks, the histone deacetylase inhibitor trichostatin-A, could stimulate photoreceptor regeneration after injury in adult mice [48]. Importantly, the regenerated cells responded to light [48], demonstrating functional recovery. Nevertheless, this mechanism of regeneration did not involve MC proliferation [48] and rather suggested direct transdifferentiation of MCs into retinal neurons, which could possibly lead to MC depletion. This could be overcome by the combined overexpression of *Ascl1* and *Lin28*. Lin28a is an RNA binding protein expressed in response to retinal injury in zebrafish [93] but not in mice [46]. *lin28a* expression is also necessary for retinal regeneration in zebrafish [93]. Combined *ascl1a* and *lin28a* overexpression mimics a regenerative response in the zebrafish retina without injury [46]. While *ascl1a* or *lin28a* expression alone does not impact the retinal phenotype, their combination induces MC proliferation and differentiation into several types of retinal neurons [46]. In the NMDA mice model of retinal injury, *Ascl1* and *Lin28* co-overexpression enhances MC proliferation in young mice [46] compared to *Ascl1* overexpression alone [92]. These studies taking advantage of the regenerative capacities of zebrafish revealed that Ascl1 and Lin28 are pieces to unlock the regeneration potential of mammalian MCs. 

## 3. Conclusions

**Figure 1:**
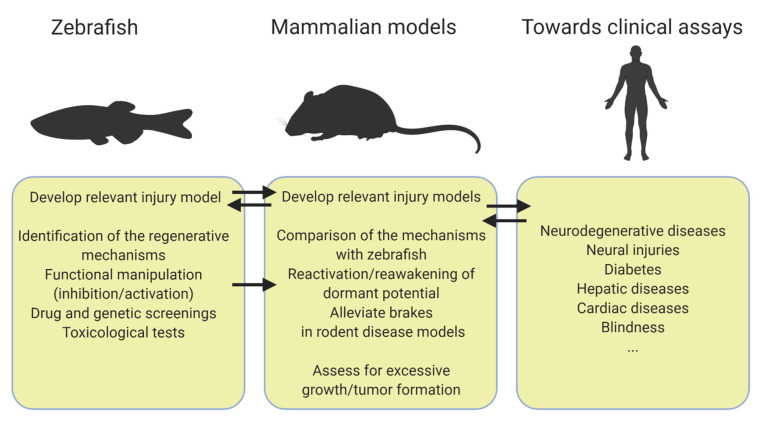
Workflow from zebrafish to mammals. Created with Biorender.com.

A question often asked to biomedical researchers using the zebrafish as a model organism is how a fish can help patients. Many studies point out that the zebrafish anatomy and physiology share many features with mammals and this is exemplified by the rapid expansion of zebrafish disease models. This review seeks to bring an answer to how zebrafish could benefit regenerative medicine by emphasizing the transposable potential of the zebrafish regenerative abilities. All the studies highlighted here share a common workflow (Figure 1) such as drug and genetic screenings to enable the identification of regulators of regeneration first in zebrafish [28,31,32,33,43]. An important trait of these studies is the versatility of a few zebrafish injury models that allow us to tackle different regenerative processes. It is for example the case of the pancreas and the liver where the zebrafish NTR model is almost exclusively employed in contrast to the various mice models of injury that are used to cover regeneration from different cellular sources (Table 1) [31,33,67]. The NTR model is also exploited to study regeneration in the heart and the brain and is continuously improving [94,95,96,97]. Although this relatively simple model provides valuable clues about regeneration, zebrafish models recapitulating more closely the disease will determine how zebrafish regenerate in such settings, therefore further increasing our understanding of regeneration and the success of transposition to mammals. A critical step of the workflow is to choose the most relevant mammalian model of injury to further explore the zebrafish discoveries (Table 1). Another key aspect is to ensure that modulating the mechanisms identified in zebrafish can improve the function of regenerated cells in vivo in mammal models.

Altogether, these studies support the idea that regenerative mechanisms are relatively well conserved even in species with low regenerative capacities, but they are repressed. It can be assumed that mammals have evolved in a way to safeguard against hyper-proliferation to prevent tissue overgrowth and tumorigenesis while maintaining functionality. In this respect, polyploidization is a common feature of many mammalian tissues during aging, homeostasis and cancer. Polyploidy has also emerged to play a role in heart regeneration [18]. Similar to cardiomyocytes, many cell types in mammals such as hepatocytes also become polyploid after birth. Although the significance for liver regeneration is poorly understood, it is likely to play a role [98,99]. It would be interesting to assess the effect of polyploidy in hepatocyte regeneration in zebrafish.

**Figure 2:**
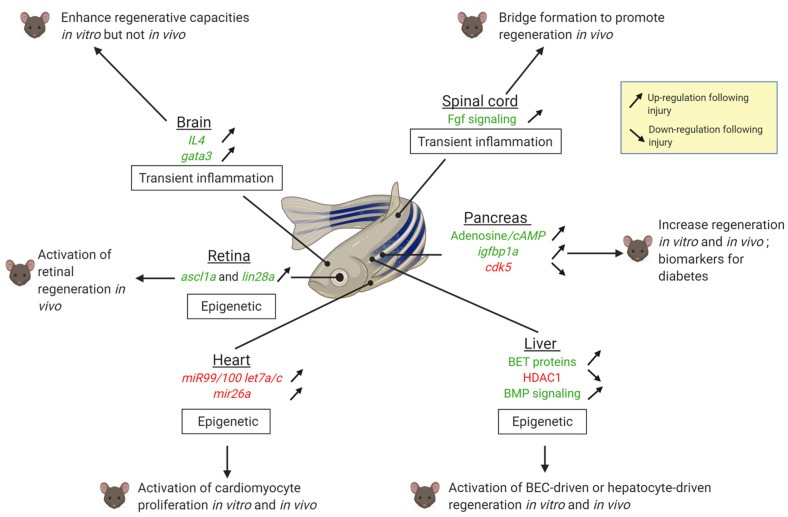
Summary of regenerative mechanisms identified in zebrafish which are able to awake the regenerative potential in mammals in the brain, the spine, the retina, the pancreas, the liver and the heart. The up-headed (vs. back-headed) arrows mean that the expression is upregulated (vs. downregulated) in zebrafish after injury. Factors highlighted in green exert positive effect in regeneration, those in red impair regeneration. Created with Biorender.com.

Two other major types of obstacles involve the immune system/inflammation and epigenetic regulations (Figure 2). Repressing a prolonged inflammatory response improves regenerative responses in the mammalian brain or spinal cord. The immune system/inflammatory response differs between organisms able to regenerate and those which cannot [100]. In organisms unable to regenerate, the immune response is generally sustained. On the other hand, the zebrafish immune response is transient, as observed in the heart [101,102] and the spinal cord [74]. This environment favors a proper regenerative response without scarring. Another obstacle to regeneration is the epigenetic repression or the loss of enhancers of pro-regenerative genes [103] as it is the case in the heart, the liver and the retina (Figure 2).

In addition to help decipher the mechanisms of regeneration, the studies performed in zebrafish also illustrate its great amenability to preclinical drug testing. To promote tissue repair, transplantation-free (or cell-free) therapies rely on administration of soluble factors, vesicles or microRNA that can be first tested in zebrafish for their efficiency and toxicity.

Even if the path is still long before we are able to overcome these obstacles and to offer beneficial treatments to patients, the zebrafish is a powerful model to help elucidate universal mechanisms of regeneration and to give clues about how and why more complex vertebrates erected barriers dampening this potential. The versatility of zebrafish enables the development of innovative models of regeneration and of novel technologies such as scRNAseq associated with CRISPR/Cas9 barcode editing for fine cell lineage tracing [104] and a growing number of genetic and metabolic reporter tools enabling non-toxic and non-invasive in vivo imaging to follow organ reconstruction and functional recovery. Associated with its regenerative capacity, all these assets confer the zebrafish with undeniable advantages over other preclinical models that will certainly accelerate research in regenerative medicine. 

## List of Acronyms

AD	Alzheimer’s disease
APAP	N-acetyl-p-aminophenol
ASCL1	Achaete Scute-Like family bHLH transcription factor 1
BECs	Biliary Epithelial Cells
BET	Bromodomain and Extra-Terminal proteins
BMP	Bone Morphogenetic Protein
CDE	Choline-Deficient Ethionine-supplemented
CNS	Central Nervous System
DTA	Diphtheria Toxin Subunit A
DTR	Diphtheria Toxin Receptor
FGF	Fibroblast Growth Factor
HDAC1	Histone Deacetylase 1
Igf	insulin-like growth factor
igfbp1	Igf binding-protein 1
IL4	interleukin-4
LEN	lepb-linked enhancer
LPCs	Liver Progenitors Cells
MCs	Müller cells
miRNAs	micro RNAs
NECA	50-N-ethylcarboxamidoadenosine
NMDA	N-methyl D-Aspartate
NTR	Nitroreductase
PDL	Pancreatic Duct Ligation
PIAA	(E)-3-(3-phenylbenzo[c]isoxazol-5-yl) acrylic acid
STZ	StrepToZotocin
T1D	Type 1 Diabetes
T2D	Type 2 Diabetes
